# Low Praziquantel Treatment Coverage for *Schistosoma mansoni* in Mayuge District, Uganda, Due to the Absence of Treatment Opportunities, Rather Than Systematic Non-Compliance

**DOI:** 10.3390/tropicalmed3040111

**Published:** 2018-10-08

**Authors:** Moses Adriko, Christina L. Faust, Lauren V. Carruthers, Arinaitwe Moses, Edridah M. Tukahebwa, Poppy H. L. Lamberton

**Affiliations:** 1 Vector Control Division, Ministry of Health, Plot 15 Bombo Road, P.O. Box 1661, Kampala, Uganda; adrikomoses@gmail.com (M.A.); moses0772359814@gmail.com (A.M.); edmuheki@gmail.com (E.M.T.); 2 Institute of Biodiversity, Animal Health and Comparative Medicine and Wellcome Centre for Molecular Parasitology, University of Glasgow, Glasgow G12 8QQ, UK; l.carruthers.1@research.gla.ac.uk

**Keywords:** Mayuge, MDA coverage, praziquantel, *S. mansoni*, systematic non-compliance, treatment-opportunities, Uganda

## Abstract

The World Health Organization (WHO) recommends praziquantel mass drug administration (MDA) to control schistosomiasis in endemic regions. We aimed to quantify recent and lifetime praziquantel coverage, and reasons for non-treatment, at an individual level to guide policy recommendations to help Uganda reach WHO goals. Cross-sectional household surveys (*n* = 681) encompassing 3208 individuals (adults and children) were conducted in 2017 in Bugoto A and B, Mayuge District, Uganda. Participants were asked if they had received praziquantel during the recent MDA (October 2016) and whether they had ever received praziquantel in their lifetime. A multivariate logistic regression analysis with socio-economic and individual characteristics as covariates was used to determine factors associated with praziquantel uptake. In the MDA eligible population (≥5 years of age), the most recent MDA coverage was 48.8%. Across individuals’ lifetimes, 31.8% of eligible and 49.5% of the entire population reported having never taken praziquantel. Factors that improved individuals’ odds of taking praziquantel included school enrolment, residence in Bugoto B and increasing years of village-residency. Not being offered (49.2%) and being away during treatment (21.4%) were the most frequent reasons for not taking the 2016 praziquantel MDA. Contrary to expectations, chronically-untreated individuals were rarely systematic non-compliers, but more commonly not offered treatment.

## 1. Introduction

Schistosomiasis is a severe, debilitating, neglected tropical disease that is often associated with poverty [1]. *Schistosoma* parasites are transmitted in areas with limited infrastructure and minimal access to, or use of, improved water, sanitation and hygiene (WASH) facilities [2,3]. The disease is endemic in 78 countries, causing an estimated 1.864 million disability adjusted life years lost (DALYs) in the over 250 million people infected, of whom >90% live in sub-Saharan Africa [4,5,6,7]. Improvements in diagnostics show that prevalence may be even higher than previously thought [8].

Currently, schistosomiasis control in endemic regions focuses heavily on regular praziquantel mass drug administration (MDA), which aims to prevent morbidity in later life by reducing infection intensities and prevalence [9]. The World Health Organization (WHO) recommends community-wide MDA in areas where prevalence in school-aged children (SAC; enrolled and non-enrolled children between the ages of 5–14) is >50% [9]. To achieve WHO goals to reduce schistosomiasis morbidity by 2020, countries need to reach targets of at least 75% preventive chemotherapy (PC) coverage of SAC and at-risk adults annually in these highly-endemic communities [10]. At-risk adults range from entire communities living in endemic areas to special groups (i.e., occupations involving frequent contact with infested water such as fishermen or irrigation workers). Models show that high PC coverage in both SAC and at-risk adults may be required for up to 15 years for successful morbidity control [11]. Models highlight that the required duration of annual MDA depends heavily on baseline intensity and the proportion of systematic non-compliance versus untreated people being randomly distributed each year [11]. However, there are few data regarding treatment across an individual’s lifetime and how non-treatment is distributed across communities [12]. Annual MDA has been successful at reducing the prevalence and intensity of infections in several areas [13,14,15,16], but hotspots remain in others [13,17]. Preventive chemotherapy is the mainstay component of schistosomiasis control, but other supportive strategies such as provision of safe water and adequate sanitation, hygiene education and snail control will be essential for the achievement of the WHO 2020 goal of elimination as a public health problem.

Coverage data at country and regional levels is often aggregated across implementation units (IUs) [12,18], whereas schistosomiasis transmission is highly focal [19]. Data on geographic coverage helps to improve tracking towards WHO goals, but there is a gap in our understanding of MDA coverage across time within individuals. Crucially, current policies are based on mathematical models that assume that coverage is homogeneous across the landscape and individuals are randomly treated each time [20]. Recent research highlights the importance of systematically untreated individuals in maintaining transmission: the effect of this group is exacerbated when overall treatment coverage is low and parasites are long-lived (i.e., *Schistosoma mansoni*) [21,22]. These models demonstrate that these untreated individuals can maintain transmission in low endemic communities even after 20 rounds of MDA [22], highlighting the importance of identifying if, and how many, individuals are untreated across their lifetime.

In 2002, Uganda was the first country in sub-Saharan Africa to begin schistosomiasis MDA. Out of 122 districts in Uganda, urogenital schistosomiasis (caused by *Schistosoma haematobium*) is found in four districts, and intestinal schistosomiasis (caused by *S. mansoni*) is found in 82 districts (synonymous with IUs in Uganda) [23,24]. In Uganda, 11.6 million people are infected, with 16.7 million at risk of infection [23,24], representing nearly 6.0% of the global population requiring schistosomiasis PC [25]. In 2018, 46 districts will receive praziquantel MDA through the national control programme [24].

Few previous studies have evaluated praziquantel coverage at an individual level or reasons for non-treatment [12,26,27,28,29,30]. In a study in 17 villages across Mayuge District, Uganda, an average coverage of 56.7% was reported, with a range of 10.9–86.6%, whilst compliance ranged from 67.8–99.1% [26]. Predictors for not being treated included Islamic religion, being a minority tribe and having lived in the village for longer [27]. Untreated individuals were also more likely to have lower socioeconomic markers such as having a low home quality, drinking unpurified water, having no home latrine and not being socially linked to people in village governments [27]. Social and physical proximity networks were also shown to be important predictors of praziquantel coverage and compliance within Uganda [26]. Similar low coverages were also observed in Côte d’Ivoire, with only 27.7% and 52.3% of people treated across two communities despite the study using door-to-door praziquantel administration [30]. Reasons reported for not taking praziquantel during that treatment round included being busy with agricultural activities, the bitter taste of the drug and/or previous adverse side effects [30]. Mixed methods studies on Koome Islands, Uganda, reported 44.7% coverage, with people more likely to have taken the last MDA if they had knowledge about schistosomiasis and general health education [28]. In this study, drug shortages and loss of health workers were identified as reasons for low coverage [28]. Higher coverages have been reported in Zanzibar, particularly in school-based treatments; however, reasons for not taking the last praziquantel still included being absent during MDA, not being visited by a drug distributor, being busy, fear of adverse events or feeling healthy [29]. Studies on other neglected tropical diseases controlled by MDA have also reported multiple complex reasons for low coverage and non-compliance [12,31]. Despite these studies on the coverage of recent praziquantel and reasons for not taking it, data on lifetime coverage and systematic non-compliance remain limited for schistosomiasis treatment [12].

We undertook household surveys of praziquantel coverage in two villages of Mayuge District, Uganda, to test model assumptions and better inform control interventions. We collected comprehensive data on praziquantel coverage, socio-economic indicators and individual-level factors that could influence praziquantel uptake. Our specific objectives were to (1) quantify praziquantel coverage, (2) determine reasons for not taking praziquantel during the last MDA round of treatment and (3) identify socio-economic and individual factors that influence praziquantel uptake in (a) the past year and (b) over a person’s lifetime. The goal was to identify and quantify drug coverage and reasons for non-treatment during recent MDA praziquantel drug coverage at an individual level. The findings may help to guide future research, interventions and targeted policy recommendations to help Uganda and other countries reach WHO 2020 goals.

## 2. Materials and Methods

### 2.1. Ethical Approval and Study Setting

The methods used in this study were reviewed and approved by the Vector Control Division Research Ethics Committee (VCDREC/062), the Uganda National Council of Science and Technology (UNCST-HS 2193) and the University of Glasgow Medical, Veterinary and Life Sciences Research Ethics Committee (200160068).

The study was conducted in two villages in Mayuge District, Uganda: Bugoto A and Bugoto B. These communities are on a peninsula on the shores of Lake Victoria approximately 25 km from the Mayuge District Headquarters, where MDA distribution is overseen. The area is a *S. mansoni* hotspot, with prevalence >90% in primary-school children [32,33]. Mayuge District was one of the first 38 IUs where MDA was carried out in 2003. From 2003 to date, there was an annual MDA administered at the district level. Interventions are implemented through the local government structure managed by the district health officer. The district health officer oversees the district health teams. These teams are responsible for the training of teachers and community medicine distributors to implement MDA; the teams also supervise them and provide a progress report [15,34]. Interventions for schistosomiasis control in Uganda using MDA began to be rolled out in 2003 in 38 of the 56 IUs with the support of the Schistosomiasis Control Initiative [35]. This has since expanded to the treatment of over 4.5 million SAC and at-risk adults annually [36], with significant reductions in *S. mansoni* prevalence, intensity and associated morbidity [37,38].

### 2.2. Survey Methodology

We conducted a census-style survey across both villages over a four-week period beginning in February 2017 (Figure S1). Before interviews began, an advocacy meeting was held with Mayuge District officials to discuss the study and obtain district approval and support. Meetings were held with the local council, chairpersons from both villages and the village health teams (VHTs). During mobilization meetings, community members were asked to provide information when the researchers arrived in their homes, but were informed that they had no obligation to do so. Households were visited door-to-door; if residents were absent, teams returned to the house at least twice for follow-up. Informed and written consent from the head of household and adults was obtained. The study respondents included any individual present in the home and above the age of 16. Both head of household and respondents were noted in the interview; however, they did not have an effect on the answers given. The main interviewers were the Ministry of Health Vector Control Officers or the District Health Drug Distributor. Each team had a member of the village health team from the respective village. There were three teams performing interviews; team members rotated between groups to ensure uniform questioning across the community.

### 2.3. Outcomes and Explanatory Variables

Our main outcomes of interest were individual praziquantel uptake in the most recent MDA (October 2016) and across an individual’s lifetime. We focused on treatment coverage (proportion of MDA target population (≥5 years) treated), but epidemiological coverage (proportion of total population) is also reported and clearly specified. Eligible individuals in this context (i.e., target population in this high endemic region) are those that are aged 5 years or older. If an individual recalled praziquantel treatment, the location of treatment was recorded. Individuals were asked to provide a reason if they were untreated in the recent MDA. Households and individual data were collected as explanatory variables for treatment coverage. We collected household information on religion, bed ownership, mosquito net ownership, house structure (materials of floor, walls and roof), water sources (for drinking, bathing and washing) and latrine structure (materials of floor, walls and roof) and usage. We also asked individuals for details of their sex, age, occupation (or current school enrolment, encompassing all levels of education) and residence time in the village. Residence time was evaluated as a continuous variable (number of years), but also as a categorical variable split into: not present during MDA (0–0.75 years), short-term residence (0.76–4 years), intermediate term residence (5–9 years) and long-term residence (10 years or more).

### 2.4. Data Analysis

Household and individual survey data were cleaned and checked for consistency using custom scripts in R v. 3.2.1 [39] (Text S1). Ninety-five percent confidence intervals (95% CI) for proportions were calculated using the Agresti–Coull method [40]. Multivariate logistic mixed effects models were created with the function glmer from the lme4 package using the binomial family to evaluate two binomial outcome variables: receiving praziquantel in the last year and receiving praziquantel during lifetime. Models were created for the target population (≥5 years). Models were created with all possible explanatory variables initially; then, insignificant predictors were removed in a stepwise manner. Best-fit models were selected using the Akaike information criteria (AIC) [41]. Adjusted odds ratios (aORs) were calculated for the best-fit multivariate models, and the Wald method was used to calculate 95% CI.

## 3. Results

We conducted 681 household interviews encompassing 3208 individuals. This covered 90.8% (95% CI: 88.0–93.0%) of Bugoto A and 90.8% (86.2–94.0%) of Bugoto B households according to estimates from Uganda’s 2014 census. According to 2014 census population projections for 2017, data were collected from 92.4% (91.3–93.5%) of individuals in Bugoto A and 92.1% (90.4–93.5%) of individuals in Bugoto B. The mean age of individuals was 17.3 years, and median age was 13 years. The main economic activities in the area were linked to fisheries, farming and small-scale business (Figure S2).

### 3.1. Self-Reported Praziquantel Uptake in Bugoto A and Bugoto B

**Figure 1:**
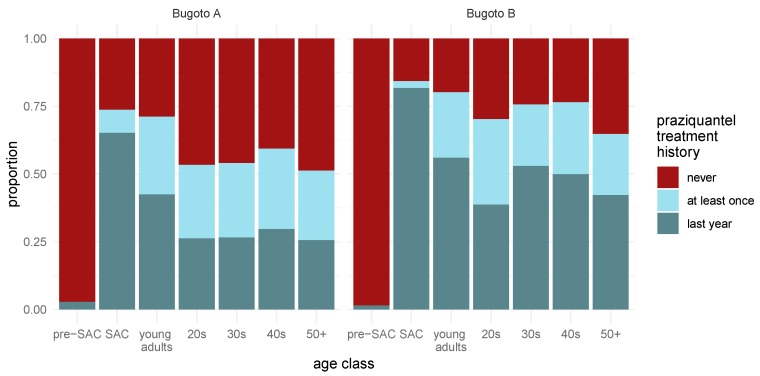
Praziquantel treatment across lifetime. The proportion of individuals in each age class that recalled receiving praziquantel treatment in 2016 (dark blue), not in 2016, but at least once in their lifetime (light blue), or never (red) are shown. For the ease of visualizing data, individuals were grouped into age groups: children were classified into pre-school-aged children (pre-SAC) (0–5 years) and SAC (6–14 years), whereas individuals who were 15 years and older were classified either as young adults (15–19 years) or by decade. Bugoto A is shown on the left, whereas Bugoto B is on the right.

Amongst surveyed individuals, 77.2% (75.7–78.6%) were eligible to receive praziquantel PC (five years or older). In the last MDA, treatment coverage was 46.5% (44.5–48.5%), whereas lifetime MDA treatment coverage was higher at 64.7% (62.8–66.7%). This means that 35.3% (33.4–37.2%) of the target population and 50.1% (48.4–51.9%) of the entire population reported never taking praziquantel. Treatment coverage among age groups in the last MDA ranged from 11.7% (eligible pre-SAC; 7.3–17.8%) to 70.7% (SAC; 67.6–73.6%) (Figure 1). If eligible pre-SAC are omitted (coverage is the same as in the last year), lifetime MDA praziquantel coverage within age groups ranged from 57.3% (20s; 52.7–61.9%) to 77.3% (SAC; 74.4–80.0%) (Figure 1).

### 3.2. Self-Reported Reasons for Not Receiving Praziquantel during the Last MDA Round

**Figure 2:**
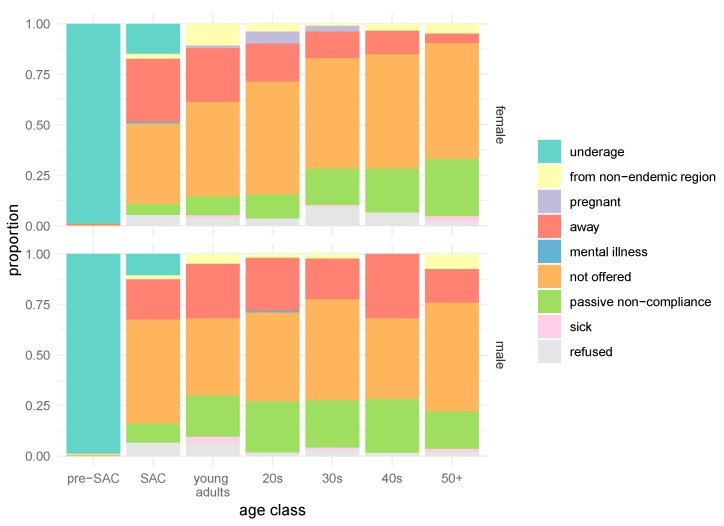
Reasons for not taking praziquantel in 2016. The proportion of individuals that reported a specific reason for not taking praziquantel is shown for both females (top) and males (bottom). For the ease of visualizing data, individuals were grouped into age groups: children were classified into pre-school-aged children (pre-SAC) (0–5 years) and SAC (6–14 years), whereas individuals who were 15 years and older were classified either as young adults (15–19 years) or by decade.

People offered nine reasons for not taking praziquantel in the last MDA (Figure 2, Table S1). The most common reason was that they were not offered treatment (44.1%; 41.3–46.9%); this included many individuals who had not known about the MDA. Absence during MDA was also common, with 19.2% (17.1–21.6%) of respondents giving this as a reason. Only 4.1% (3.1–5.4%) of respondents indicated fear of side effects/active treatment refusal as the reason for not taking praziquantel.

### 3.3. Socio-Economic and Individual Risk Factors Influence Praziquantel Uptake

**Figure 3:**
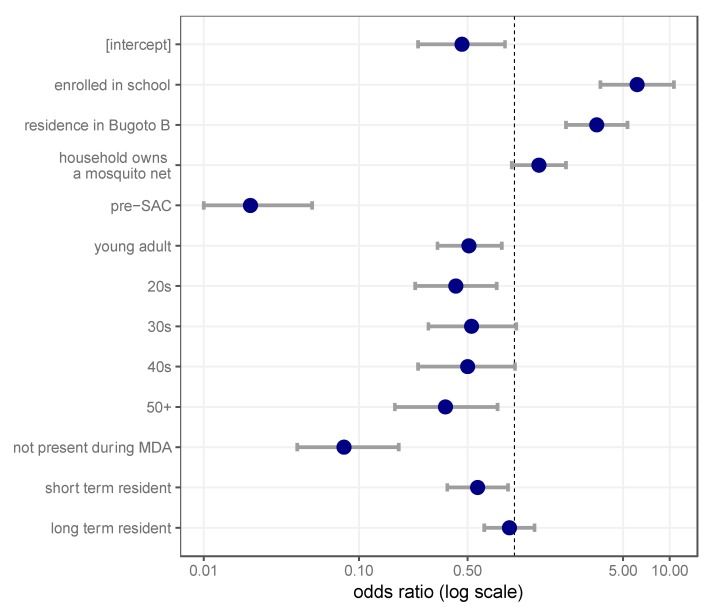
Multivariate analysis of socio-economic and individual factors that influence praziquantel uptake in the last year. Individuals were grouped into age groups: children were classified into pre-school-aged children (pre-SAC) (0–5 years) and SAC (6–14 years), whereas individuals who were 15 years and older were classified either as young adults (15–19 years) or by decade. The intercept represents an unenrolled SAC that resides in Bugoto A without a mosquito net and has lived in the village an intermediate time (5–9 years). Adjusted odds ratios are plotted on a log scale, with coloured dots indicating the estimate, and grey lines indicate 95% CI for each estimate.

Mixed effects models were used to investigate significant predictors of praziquantel treatment during MDA amongst the eligible population (five years and older). The best-fit model for taking praziquantel in 2016 included age group, residence time (as a categorical variable; see Materials and Methods), village residence, school enrolment and household ownership of a mosquito net (Figure 3). There were very similar predictors for lifetime praziquantel treatment (Figure S3). In all models, household identity was included as a random effect.

Specifically, the odds of an individual receiving praziquantel in 2016 was highest in SAC, with the majority of age groups having significantly lower odds: pre-SAC (adjusted odds ratio (aOR): 0.02, 95% CI: 0.01–0.05), young adults (aOR: 0.51, 95% CI: 0.32–0.83), 20s (aOR: 0.42, 95% CI: 0.23–0.77), 30s (aOR: 0.53, 95% CI: 0.28–1.03), 40s (aOR: 0.50, 95% CI: 0.24–1.01) and 50s (aOR: 0.36, 95%CI: 0.17–0.78). Enrolment at a school, including primary, secondary or any graduate education, increased odds of taking praziquantel (aOR: 6.17, 95% CI: 3.58–10.64), as did residing in Bugoto B (aOR: 3.39, 95% CI: 2.15–5.35). Having a mosquito net at home could increase the odds of taking praziquantel in the last year (aOR: 1.44, 95% CI: 0.96–2.15). Although this predictor was not significant at *p* = 0.05, including this variable improved the fit of the model and was retained for the final model. Residence time was also an important factor in determining whether an individual received praziquantel in the last year. Intermediate residence time (5–9 years) was similar to long-term residence (aOR: 0.93, 95% CI: 0.64–1.35). However, if individuals were not living in Bugoto during the previous MDA campaign, their odds of receiving treatment elsewhere were low (aOR: 0.08, 95% CI: 0.04, 0.18), and short-term residence also decreased the odds of receiving praziquantel (aOR: 0.58, 95% CI: 0.37, 0.91). There were no significant interactions between the variables in this model.

Predictors of taking praziquantel treatment across lifetime were similar to the last year, but the direction and magnitude of the predictors often varied (Figure S3). However, mosquito net ownership was no longer included in the best fit model, but there was a significant interaction between age and residence time. The odds of receiving praziquantel across one’s lifetime increased with school enrolment (aOR: 3.62, 2.13–6.15), residence in Bugoto B (aOR: 2.23, 1.53–3.24) and residence time in the village (for each year, aOR: 1.38, 1.29–1.47). Unlike praziquantel in the last year, most adults had higher odds of being treated with praziquantel than children (YA, aOR: 3.22, 1.40–7.39; 20s, aOR: 6.06, 2.83–12.97; 30s, aOR: 10.12, 4.37–23.40; 40s, aOR: 10.91, 4.37–23.40). Pre-SAC were less likely to receive praziquantel compared with SAC (aOR: 0.04, 0.00–0.61), whereas older (50s) individuals had similar odds as SAC (aOR: 2.29, 0.72–7.29). There was a significant interaction between age and residence; often times reducing the odds of taking praziquantel (i.e., residence years* 20s (interaction denoted by *), aOR: 0.77, 0.72–0.82).

## 4. Discussion

Our results highlight that annual praziquantel uptake rates are well below the WHO targets in Mayuge District, Uganda. Treatment coverage during the most recent MDA (2016) was only 46.5% of eligible residents, and 35.3% of eligible residents reported having never taken the drug. To achieve the WHO’s goal of controlling schistosomiasis morbidity by 2020 and ultimately eliminating it as a public health problem, at least 75% treatment coverage is needed annually for over 10 years [18]. A key assumption of this goal is that the majority of individuals untreated each year are randomly distributed. Therefore, as annual MDA is repeated, untreated people are ‘mopped up’. This would result in the number of people who have never received praziquantel diminishing over time. In our study communities, in a country where MDA has been going on for longer than all other sub-Saharan African countries, a third of eligible individuals reported that they have never taken praziquantel. These untreated individuals are not randomly distributed and are at risk of chronic schistosomiasis and associated morbidity. They also act as reservoirs for reinfection of treated individuals [21,22].

Low MDA treatment coverage, annually and over an individual’s lifetime, has serious implications for national disease control and prevention programmes and the sustainable control of schistosomiasis morbidity and transmission. The self-reported uptake in our villages was on the lower end of coverage compared to other studies, especially amongst adults [26,27,28,29,42,43]. Adult coverage in 2016 was 34.8% (95% CI: 32.3%, 37.3%), whereas 70.7% (95% CI: 67.6–73.6%) of SAC were treated. Levels were similar on Koome Island, Uganda (44.7%, 95% CI: 40.8–48.7% ), but islanders have been identified as national priorities for improved interventions [28]. To the authors’ knowledge, only one published study in sub-Saharan Africa has observed lower treatment uptake at 28.2% (95% CI: 22.9, 33.6%), in the city of Jinja, Uganda [44], where advocacy was lacking.

Across Uganda, only 1/3 of the population requiring PC was treated in 2016, partly due to some completely untreated districts [36]. Mayuge District has previously reported low praziquantel coverage in 2009 (35%) [45], but attributed it to a lack of praziquantel stock. Our study focused on two geographically-close communities, but data reflect similar trends across the district and country since the control programme began.

The majority of studies have focused on recent treatment coverage [26,28,29,42,43,46], but have not addressed lifetime treatment data and the important issue of systematic non-treatment rates [11,12]. We show that a third of the at-risk population has never been treated despite 14 years of MDA. Using these data to parameterise models could have a significant effect on the predicted duration that repeated MDA is needed to control morbidity. Using just our coverage rates of 70.7% in SAC and 34.8% in adults, the sensitivity analyses by Turner and colleagues (2017) indicate that MDA is needed for 6–9 years with a 20% systematic non-compliance [11]. Our finding of 35.3% systematically non-treated eligible individuals, and 49.5% of the entire population never receiving treatment, likely explains the high infection prevalence and intensity still observed in these communities after 14 years of MDA [33,47]. If these low annual treatment rates and high lifetime MDA non-treatment continue, MDA programmes in districts such as this may potentially never control morbidity.

Despite low coverage, several individual and community factors were significantly correlated with praziquantel uptake. Current school enrolment status significantly increased the chance of receiving praziquantel, both within primary school children and adults enrolled in secondary and tertiary education. Residence in Bugoto B increased chances of praziquantel treatment in the last year and over lifetime. Bugoto B has a smaller total population and is more spread out than Bugoto A. This potentially makes it easier for distributors in Bugoto B to keep track of who has, and has not, received treatment and possibly makes it more likely to identify and include newcomers in the MDA. The higher praziquantel uptake may also be a product of established and stable social networks in Bugoto B; these social ties have been shown to be important in other settings [26]. Bugoto B has more long-term residents (residents have spent an average of 69% of their life in the village) compared to Bugoto A (average of 56% of their life in the village). In our study, the longer an individual had lived in the village, the more likely he/she was to receive treatment, especially across a person’s lifetime (but this is slightly mitigated by residence time). Lastly, mosquito net ownership can increase the likelihood of taking praziquantel in the last year, which could be interpreted as a proxy for health-seeking behaviour. These factors are important to consider when modifying programmes to increase praziquantel uptake.

The term systematic non-compliers is often used instead of systematically non-treated. Praziquantel MDA coverage can be significantly lower than other MDA programmes locally and nationally and is commonly attributed to the fear of side effects [48]. Despite this common belief, residents in our study were significantly more likely to have not been offered praziquantel, to have been away during treatment or to have been passive non-compliers than to have actively refused it. Of the target population that had not taken the drug in the last MDA, only 4.6% refused treatment. This is similar to other praziquantel studies [29,49] indicating that the belief that fear of side effects reduces treatment uptake is unfounded and unhelpful.

The most common reason for not taking praziquantel in the last year was not being offered, with rates much higher than other studies [29]. Increasing the number of community drug distributors (CDD) could increase drug coverage. In these villages, each drug distributor covers 150 households, well above the recommended 25–30 households [50]. If each CDD is responsible for fewer households, MDA coverage increases significantly [45,51]. Encouraging house-to-house administration in these communities should also increase coverage [45,46]. In general, most adults taking praziquantel in the last year received treatment at central locations rather than their household. However, a higher proportion of Bugoto B residents compared to Bugoto A residents reported receiving praziquantel treatment at home in 2016, which may partly explain the higher coverage in Bugoto B. The second most common reason for not taking praziquantel was being away during MDA, compounded by the absence of praziquantel in almost all Ugandan frontline health facilities. Similar findings were reported for lymphatic filariasis, where MDAs were reported to be too short and not lasting long enough to reach the whole communities [31]. Leaving drugs at health centres or with key personnel after each MDA may enable a significant mop up of these mobile populations. Many reasons given for not taking praziquantel in the last MDA suggest that improved educational campaigns and effective mobilization could increase health-seeking behaviour and improve community-wide MDA. A recent study supports these conclusions with findings that anytime was suitable for treatments if people were informed in advance [30]. They also reported that knowledge of the disease was a positive predictor of treatment, highlighting that communication is key at all levels. In addition, the dry season was suggested as the best time of year to go with people less likely to be in the fields and absent during treatment than in the rainy season. They further found that in a large village, house-to-house treatment was preferred, but this was not important in a smaller village where people possibly all lived closer to a drug distribution point [30]; this could also further support our findings of higher praziquantel success in the smaller population of Bugoto B, although this is a geographically dispersed population.

Anecdotal evidence during our surveys suggested that individuals believed they were cured after one round of treatment and therefore did not take praziquantel in subsequent MDAs (i.e., passive non-complier). In Côte d’Ivoire, three-quarters of people treated during the study said they would not take the drug again, despite a significant proportion of people saying they felt better after treatment [30]. In Zanzibar, treatment fatigue was reported [29], which also may explain our interaction between age and duration in the village and the reduction of treatment uptake in the last MDA. Others believe praziquantel MDA is for SAC only or had simply never heard of the MDA. Studies in Nigeria show that obtaining community support and involvement before praziquantel MDA implementation contributes to an effective treatment strategy for schistosomiasis [52]. However, knowledge of schistosomiasis transmission and prevention that increases the likelihood of taking praziquantel MDA has not successfully reached the intended communities as suggested [42].

The key limitations of this study were that only two villages were surveyed and that the source of data on participants’ past treatment with praziquantel was through interviews, which may be prone to recall bias. Participants might not be in a position to differentiate praziquantel from other medicines they have taken, and this might be exacerbated by the time between the most recent MDA and the survey date. This was minimised where possible by showing images of praziquantel and describing its bitter taste to the interviewees. Thus, if individuals were to confuse treatments with other MDA programmes, it would likely result in a higher recall of the wrong drug rather than no recall at all. If people had taken the drug many years before and forgotten, then this could lower the lifetime coverage, which could be better inferred as coverage within memory. In addition, MDA has only been occurring in Mayuge for 14 years, and so, lifetime refers more to the lifetime of the national control programme, rather than, for example, the full extent of an adult’s life. Furthermore, if someone had taken praziquantel once a long time ago, at the start of the national programme, but is now systematically untreated, then the effect on transmission and morbidity may remain relatively unchanged if they are still being exposed in these highly endemic communities. However, similar low coverages in recent treatments have been reported across Mayuge [53], indicating that our data for recent treatment are in line with other studies and provide additional information regarding long-term treatment exposure, which other studies rarely address, despite it being highlighted by modellers as an important knowledge gap [11,12].

The communities involved in this study are incredibly diverse, and there are factors that were not measured that are also likely to influence praziquantel uptake. For example, additional household characteristics, ethnicity and social networks have also been shown to influence likelihood of praziquantel treatment [26,53]. We therefore recommend further studies into the social and cultural factors influencing long-term praziquantel uptake and schistosomiasis transmission in these communities. This will help highlight which targeted mobilization approaches are feasible and are most successful in increasing praziquantel coverage and reducing transmission.

## 5. Conclusions

Schistosomiasis is associated with low socio-economic status, poor sanitation, lack of access to healthcare systems and frequent contact with infected water bodies [54,55]. Annual MDA has been successful at reducing prevalence and intensity of infections in several areas [14,16,37,38], but hotspots remain in others [13,17]. While PC is an important component of schistosomiasis control, other supportive strategies such as provision of safe water and adequate sanitation, hygiene education and snail control will be essential for the control and elimination of schistosomiasis. However, improvements in MDA can impact disease morbidity and improve short-term outcomes for stakeholders. Based on community surveys, praziquantel uptake could be improved by leaving supplies of praziquantel at health facilities or with CDDs, improving awareness that people can get re-infected post-treatment and emphasizing that praziquantel is recommended for adults in these communities, not just children. This study supports the notion that bottlenecks for schistosomiasis control by MDA are occurring at the level of distribution and are not driven by non-compliance.

## Supplementary Materials

The following are available online at http://www.mdpi.com/2414-6366/3/4/111/s1: Text S1: Supplementary methods; Figure S1: Community survey template, Figure S2: Occupations of individuals in each village above the age of fourteen by gender, Figure S3: Multivariate analysis of socio-economic and individual factors that influence praziquantel uptake across one’s lifetime, Table S1: Self-reported reasons for not receiving praziquantel during the last MDA (2016).
