# Supplementary material for: Low Praziquantel Treatment Coverage for Schistosoma mansoni in Mayuge District, Uganda, Due to the Absence of Treatment Opportunities, Rather Than Systematic Non-Compliance
Source: Trop Med Infect Dis. 2018 Oct 8;3(4):111. doi: 10.3390/tropicalmed3040111 (PMC6306755; doi:10.3390/tropicalmed3040111)
Supplement: Supplementary file 1 [file tropicalmed-03-00111-s001.pdf]

Supplementary Materials: Low Praziquantel Treatment Coverage for *Schistosoma mansoni* in Mayuge District, Uganda, Due to the Absence of Treatment Opportunities, Rather Than Systematic Non-Compliance

Moses Adriko, Christina L. Faust, Lauren V. Carruthers, Arinaitwe Moses, Edridah M. Tukahebwa and Poppy H. L. Lamberton

Text S1. Supplementary Methods

Survey Cleaning

Fisherfolk and related occupations (coxswains, boat loaders) were combined into one high frequency water contact group. The educational category includes both students and teachers. The 'other' category includes occupations such as builders, boda boda drivers (motorbike taxis) and brick makers. All unemployed individuals with children in the household were classed as homemakers, whereas unemployed was reserved for individuals without a specific occupation or caretaker duties. Other common occupations include education-related, farmers and small-scale business (i.e., shopkeepers)

Reasons for Not Receiving Praziquantel Treatment

Individuals were not offered treatment, including individuals who did not know treatment was available, either at all or thought it was not available for adults. Individuals could be passive non-compliers, if they knew treatment was occurring, but they did not seek out treatment. Individuals could have been away during last year's MDA or have recently moved from a non-endemic region where MDA was not given. Other reasons given for not taking treatment were that they were sick, pregnant or had a mental illness. Individuals that were offered, but refused treatment were grouped with individuals that did not seek treatment because they feared drug side effects.

House no.:	________	GPS location house:	Lat:________	Long:________		
Village:	________	House Floor:	Walls:		Roof:	
Observer:	________	GPS location latrine:	Lat:________	Long:________	Shared with________.	
Date:	________	Latrine Floor:	Walls:		Roof:	

Do you sleep under a mosquito net?	yes / no	Do you sleep on the floor or a bed?		Bed/Floor	Religion:		
Does your household have a private pit latrine?		(circle one)	Please tick any appropriate. if >1, number 1 for most commonly used				
Does your household have access to a shared pit la	yes / no	(circle one)					
Is the pit latrine you use a municipal latrine?	yes / no	(circle one)					
If yes, how frequently do you use the latrine?	Always:	Sometimes:		Rarely:		Never:	
If no/infrequent/never, where do you ease yourse	Bush:	Lake:	Plantation:	Other:			
Where do you get your drinking water?	Borehole:	Lake:	Rainwater tank:	Well:	Tap:	Other:	
Where do you bathe/get water for bathing?	Borehole:	Lake:	Rainwater tank:	Well:	Tap:	Other:	
Do you bring the water home or bathe there?		Home:	There:				
Where do get water to wash your family's clothes?	Borehole:	Lake:	Rainwater tank:	Well:	Tap:	Other:	
Do you bring water home or wash clothes there?		Home:	There:				

	Name (**R-indicates respondent**)	R	Age	Sex M/F	Occupation	How long have you lived in the village?	Have you ever been treated for bilharzia?	When were you last treated?	Where were you treated?	If not 2016 why not?
**Head of house**										
**Adults (>18)**										

	Name	Age	Sex M/F	School enrolled at	Class	How long have they lived in the village?	Have you ever been treated for bilharzia?	When were you last treated?	Where were you treated?	If not 2016 why not?
**Children (<19)**										

Figure S1. Community survey template

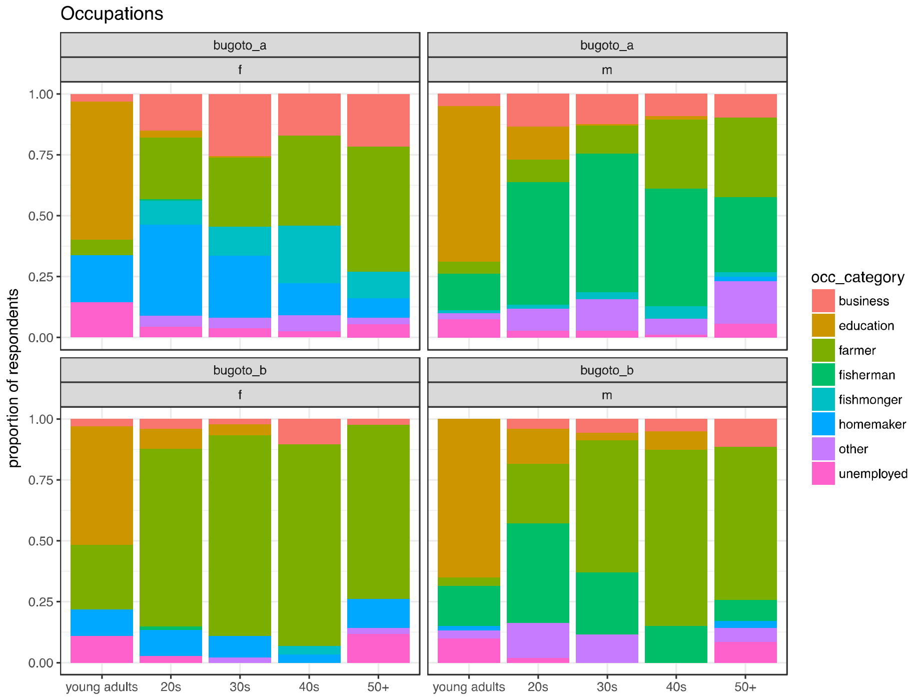

Figure S2: Occupations of individuals in each village above the age of fourteen by gender.

In Bugoto A, there is a higher diversity of occupations. In Bugoto B, most people are farmers whereas in Bugoto A the majority of males are fishermen and women have a wide variety of occupations from homemaker to business.

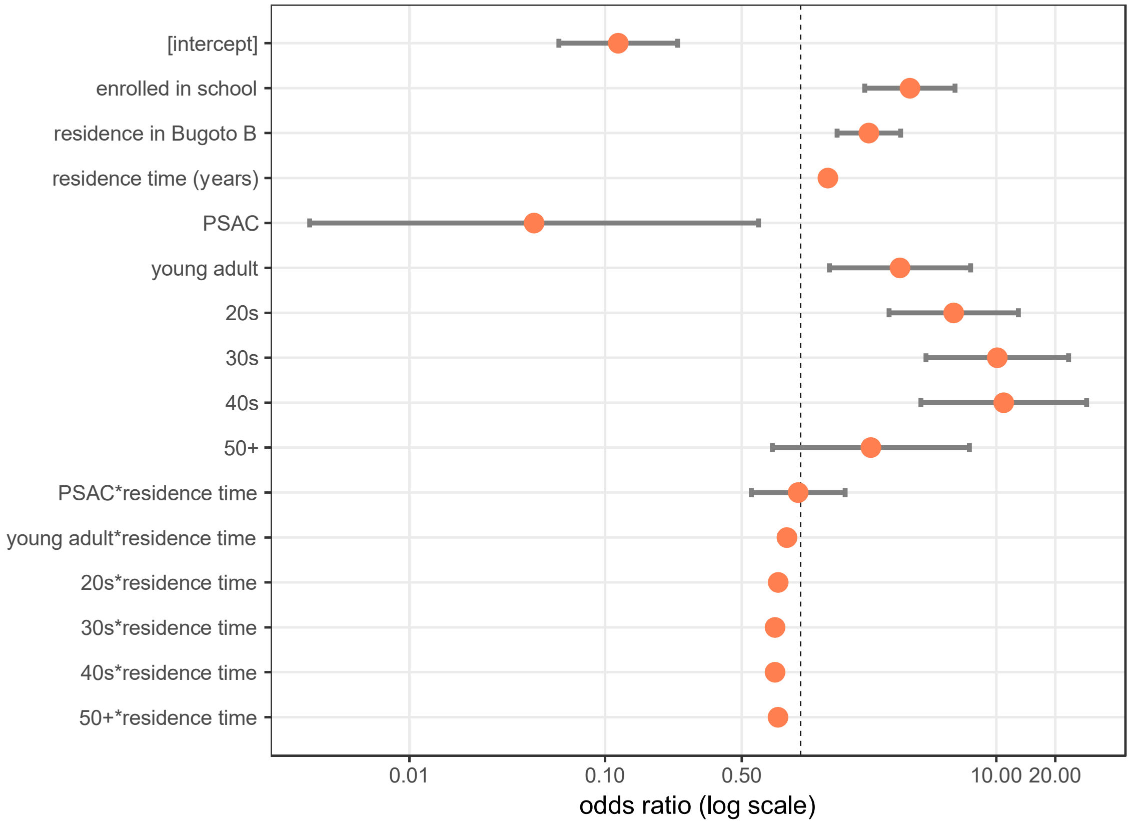

Figure S3: Multivariate analysis of socio-economic and individual factors that influence praziquantel uptake across their lifetime. The intercept represents an unenrolled SAC that resides in Bugoto A. Adjusted odds ratios are plotted on a log scale, with coloured dots indicating the estimate, and grey lines indicate 95% CI intervals for each estimate.

Table S1: Self-reported reasons for not receiving praziquantel during the last MDA (2016). The number of individuals that reported not taking praziquantel in Mayuge District's 2016 praziquantel MDA. For those that provided a reason, the number of individuals in each category for each age group is listed.

Age group		SAC (6-14)	YA (15-19)	20-29	30-39	40-49	>50	Total
did not take praziquantel in 2016 MDA		29.4 % (260/ 885)	53.7% (159/ 296)	71.6% (345/ 482)	67.3% (230/ 342)	65.9% (143/ 217)	66.9% (109/ 163)	52.2% (1246/ 2139)
reason for not taking	**underage**	30	0	0	0	0	0	30
	**from non-endemic region**	5	11	9	4	2	8	39
	**pregnant**	0	1	11	3	0	0	15
	**away**	62	43	82	39	30	12	268
	**mental illness**	1	0	1	0	1	0	3
	**not offered**	107	65	163	111	65	58	569
	**passive non-compliance**	17	22	57	46	31	22	195
	**sick**	0	3	0	2	0	2	7
	**refused**	14	7	9	13	7	2	52
